# *RPGR^ORF15^* Mutations Disrupt Lysosomal Lipid Metabolism in Retinal Pigment Epithelium Cells and Cause Retinitis Pigmentosa

**DOI:** 10.1167/iovs.66.14.61

**Published:** 2025-11-25

**Authors:** Mengmeng Ren, Xiang Chen, Pan Gao, Yukan Huang, Shanshan Yu, James Reilly, Kui Sun, Yunqiao Han, Hualei Hu, Pei Li, Jiong Luo, Liyan Dai, Yuejie Zhu, Qunwei Lu, Xinhua Shu, Shusheng Wang, Xiang Ren, Zhaohui Tang, Mugen Liu

**Affiliations:** 1 Key Laboratory of Molecular Biophysics of Ministry of Education, College of Life Science and Technology, Huazhong University of Science and Technology, Wuhan, Hubei, People's Republic of China; 2 Department of Ophthalmology, Union Hospital, Tongji Medical College, Huazhong University of Science and Technology, Wuhan, Hubei, People's Republic of China; 3 Institute of Visual Neuroscience and Stem Cell Engineering, College of Life Sciences and Health, Wuhan University of Science and Technology, Wuhan, Hubei, People's Republic of China; 4 Department of Biological and Biomedical Sciences, Glasgow Caledonian University, Glasgow, Scotland, United Kingdom; 5 Department of Cell and Molecular Biology, Department of Ophthalmology, Tulane Personalized Health Institute, Tulane University, New Orleans, Louisiana, United States

**Keywords:** retinitis pigmentosa GTPase regulator (RPGR), retinal degeneration, retinal pigment epithelium (RPE), lysosomes, lipid metabolism

## Abstract

**Purpose:**

X-linked retinitis pigmentosa (XLRP) is a severely blinding retinal disease, most of which are due to mutations in retinitis pigmentosa GTPase regulator (RPGR). The patients with RPGR mutations exhibit severe retinal pigment epithelium (RPE) atrophy and photoreceptor degeneration. Previous research mainly focused on the role of RPGR in the connecting cilia of photoreceptors. However, the mechanism underlying RPE deficiency in patients remains unclear. Moreover, the function of RPGR in RPE cells has not been investigated.

**Methods:**

To investigate the mechanisms underlying RPE atrophy and the role of RPGR in RPE cells, the *rpgra^−/−^* zebrafish, human RPE cell line RPE-1, and ARPE-19 were utilized. Histological analysis, immunofluorescence, and lipid staining were used to investigate the morphology of photoreceptor and RPE cells, as well as the accumulation of lipid droplets (LDs) in RPE cells. FITC-labeled OS were used to evaluate the engulfment and degradation capabilities of RPE cells.

**Results:**

The zebrafish homolog of human *RPGR^ORF15^*, *rpgra*, is expressed in RPE cells. The *rpgra^−/−^* zebrafish exhibits RPE atrophy, followed by photoreceptor degeneration. Loss of *rpgra* impairs lysosome formation in RPE cells, leading to defective RPE phagocytosis. This triggers lipid metabolism disorders, ultimately causing RPE and retinal degeneration.

**Conclusions:**

*RPGR^ORF15^* is essential for maintaining lysosome function and lipid metabolism homeostasis in RPE cells. This finding elucidates the previously unrecognized role of *RPGR^ORF15^* in RPE cells. This study provides new insights into the mechanisms underlying RPGR-associated retinal diseases and offers potential therapeutic approaches.

Retinitis pigmentosa (RP) is a genetic retinopathy characterized by progressive photoreceptor degeneration and retinal pigment epithelium (RPE) atrophy.^1^ Mutations in the retinitis pigmentosa GTPase regulator (*RPGR*) gene account for approximately 70% of all X-linked retinitis pigmentosa (XLRP) cases, one of the most severe forms of RP.^2^ Patients with *RPGR* mutations suffer from severe visual impairment, which is associated with retinal atrophic macular degeneration, choriocapillaris loss, and RPE atrophy.^3^ Unfortunately, the underlying molecular mechanisms are still not fully understood.

Human *RPGR* consists of 2 major isoforms: RPGR^ex1-19^ and RPGR^ORF15^.^2^ They share an N-terminal regulator of chromosome condensation like (RCC1-like) domain, but with different C-terminals (Supplementary Fig. S1). Specifically, RPGR^ex1-19^ contains a putative prenylation site at its C-terminus, whereas RPGR^ORF15^ features a repetitive glycine/glutamic acid-rich domain (named ORF15), a mutational hotspot found in patients with XLRP.^2^^,^^4^ Notably, RPGR^ex1-19^ is ubiquitously expressed in multiple tissues,^5^^,^^6^ whereas the *RPGR^ORF15^* localizes predominantly to the photoreceptors.^7^ Two dog models with different mutations in RPGR (XLPRA1 and XLPRA2) exhibited abnormal retinal function.^8^ Loss of RPGR in mice models and humans results in progressive retinal degeneration characterized by photoreceptor dysfunction.^9^^,^^10^ Zebrafish possess 2 homologs of the human RPGR gene: *rpgra*, which is homologous to human *RPGR^ORF15^*, and *rpgrb*, which has 2 transcripts (*rpgrb^ex1-17^* and *rpgrb^ORF15^*), analogous to the human *RPGR^ex1-19^* and *RPGR^ORF15^* isoforms.^11^ Liu et al. have demonstrated that *rpgra* is highly expressed in adult zebrafish eyes, whereas *rpgrb^ORF1^*^5^ expression is the lowest.^12^ Therefore, zebrafish serve as an excellent model to study the function of RPGR^ex1-19^ and RPGR^ORF15^. Our recent study first constructed an *rpgra* knockout zebrafish and demonstrated that *rpgra*^−/−^ zebrafish exhibited progressive retinal degeneration.^12^

Previous studies have predominantly focused on the role of RPGR in photoreceptor cells.^13^^,^^14^ However, clinical observations in patients harboring *RPGR* mutations reveal additional pathological features, including macular degeneration, RPE atrophy, and choroidal capillary loss.^3^ The genotype-phenotype correlation analysis revealed that 30 of 41 patients with RPGR mutations also exhibited RPE atrophy,^15^ suggesting that RPGR defects may directly compromise RPE structure and function, which is a plausible contributor to retinal dysfunction in these patients.^3^ At present, the pathogenic mechanism of RPE degeneration in patients with *RPGR* mutation remains poorly understood.

RPE is a monolayer of pigmented epithelial cells that are located between the choriocapillaris and photoreceptors.^16^^,^^17^ The daily phagocytosis of photoreceptor outer segments (OS) by RPE cells is indispensable for OS renewal, lipid metabolic homeostasis, and visual function.^18^ The dynamic process of phagocytosis in RPE is known as “phagocytotic flux,” including the formation of phagosomes, the fusion of phagosomes and lysosomes, and degradation of substrates within phagolysosomes.^19^ Lysosome-associated membrane protein 1 (LAMP1) is a marker for lysosome biogenesis, and cathepsin B (CTSB) and cathepsin D (CTSD) are responsible for degradation within the lysosome.^20^^,^^21^ Defective phagolysosome degradation in RPE cells leads to the accumulation of lipid droplets^22^^,^^23^ and aberrant cholesterol metabolism,^19^ which may result in lipid metabolism disorders and retinal diseases. Lysosomal dysfunction in RPE cells has been recognized as a pivotal factor in disorders such as age-related macular degeneration (AMD).^24^ Other RP genes, such as *CYP4V2*, *ABCA4*, and *APOE* have been implicated in RPE lipid metabolism defects.^25^^–^^27^ However, the role of *RPGR* in RPE remains unexplored. Crucially, no studies have investigated whether lysosomal dysfunction or RPE lipid metabolic disruptions contribute to the pathogenesis of RPGR-associated inherited retinal degenerations.

Previous studies have demonstrated RPGR expression in the photoreceptors of mice.^2^^,^^28^^–^^30^ Here, we discovered that Rpgra is also expressed in the RPE cells of zebrafish. This study demonstrated that *RPGR^ORF15^* is a novel regulator of lysosomal formation and lipid metabolism in RPE cells, which is essential for the homeostasis of retina. Loss of *rpgra* impaired lysosome biogenesis, inducing excessive lipid droplets’ (LDs) accumulation in RPE cells. This pathological cascade triggered RPE structural atrophy and subsequently progressive retinal degeneration. Our study reveals a novel pathological mechanism of RPGR-related retinal degeneration and provides potential therapeutic targets centered on RPE lysosomal lipid metabolic modulation.

## Methods

### Zebrafish Maintenance

All studies involving zebrafish followed guidelines approved by the Ethics Committee of Huazhong University of Science and Technology and conducted in accordance with the ARVO Statement for the Use of Animals in Ophthalmic and Vision Research. Zebrafish were cultured in a constant circulating water system at 28.5°C with a 14-hour light/10-hour dark cycle. Fertilized eggs were collected and maintained in E3 medium in an incubator (at approximately 28.5°C) for 72 hours until the larvae hatched.

### Hematoxylin and Eosin (H&E) Staining

Zebrafish eyes were isolated and fixed in 4% paraformaldehyde (PFA) at 4°C overnight, and then dehydrated with 30% sucrose at 4°C overnight, and finally embedded in optimal cutting temperature (OCT) compound. The procedure of frozen section was carried out as described in previous studies.^31^ Retinal sections were cut with the cryostat (Leica CM1950; Leica, Wetzlar, Germany) at 10 to 15 µm thickness. The slides were dried for 30 minutes at 37°C and then stored at –20°C. Cryosections were stained with hematoxylin and eosin (H&E; Beyotime, C0105S, China) for analysis according to the manufacturer's instructions under standard conditions. The images were acquired using an optical microscope BX53 (Olympus).

### Immunofluorescence Assay

Immunofluorescence of retinal sections and RPE flat mount eyecups was carried out as described previously.^32^^,^^33^ The sections were air dried at room temperature (RT) and incubated with PDT (PBS/1%DMSO/0.1%Triton X-100) for 10 minutes. Then, the sections were blocked for 1 hour with blocking solution (PDT/1%BSA/10%goat serum), followed by primary antibodies that were diluted in blocking buffer and added onto the slides and incubating them overnight at 4°C. The slides were washed with PBS and incubated with secondary antibodies incubated for 1 hour in 37°C. The cell nuclei were stained with DAPI (5 µg/mL) after washing with PBS three times. The sections were washed with PBS and mounted with 50% glycerol. The samples’ images were captured using a confocal laser-scanning microscope (FV3000, Olympus). The primary antibodies used in this study are listed in Supplementary Table S1.

### Nile Red, Filipin, and Lysotracker Staining

The procedure was the same as the immunofluorescence assay before blocking. Then, cryosections were washed with PBS and stained with 2 µg/mL Nile Red (N1142, Invitrogen) for 10 minutes. DAPI was used to label cell nuclei. The staining for cultured cells and RPE flat mounts was the same as described above. Filipin staining was used with a cholesterol detection kit (SAE0087, Sigma). The protocol was similar to the procedure mentioned above. After washing with PBS, the cryosections and cultured cells were stained with 10 mg/mL Filipin (SAE0087, Sigma) for 2 hours. The slides were then mounted with 50% glycerol. For lysotracker (L7528, Thermo Fisher) staining, zebrafish eyes were injected with 5 mM lysotracker for 3 hours, whereas RPE-1/ARPE-19 cells were treated with 2 µM lysotracker for 30 minutes. The slides were mounted under glass coverslips. The fluorescence images were captured with a confocal microscope (FV3000, Olympus). The size and number of LDs in the images were quantified using ImageJ software. Triglyceride (TG) and free cholesterol (FC) concentrations were determined by commercial kits (respectively, AKFA003C and AKFA001C, Beijing Box Technology Co. Ltd), according to the manufacturer's instructions, respectively.

### Transmission Electron Microscopy 

For ultrastructural analysis, zebrafish eyes at 2 and 4 months post fertilization (mpf) were collected and processed as described previously.^34^ The eyes were fixed in 2.5% glutaraldehyde (0.1 M PBS, pH 7.4) overnight at 4°C. After washing with PBS, the eyes were refixed with 1% osmium tetroxide at RT for 2 hours. Then, the eyes were dehydrated in gradient with ethanol and incubated in acetone for 20 minutes. The eyes were embedded in epoxy resin after treatment with propylene oxide. Next, the Reichert-Jung ultramicrotome was used to produce 100 nm thickness ultrathin slices that were then stained with uranyl acetate and 3% lead citrate. Images were captured through a transmission electron microscopy (TEM; HT7700, Hitachi).

### Cell Culture and RNA Interference

Human RPE-1 cells (CRL-4000, American Type Culture Collection) and ARPE-19 cells (CRL-2302, American Type Culture Collection) were cultured in Dulbecco's Modified Eagle Medium (DMEM; Gibco 11330057, Thermo Fisher) supplemented with 10% fetal bovine serum (FBS), and DMEM/F12 (Gibco 11330057, Thermo Fisher) supplemented with 10% FBS in 5% CO_2_ atmosphere at 37°C, respectively. Small interfering RNAs (siRNAs) targeting encoding regions of human *RPGR^ORF15^* and *RPGR^ex1-19^* were synthesized by RiboBio (Ruibo Biotechnology Co. LTD, China). The target sequence of *RPGR^ORF15^* siRNA (si*RPGR^ORF15^*) and *RPGR^ex1-19^* siRNA (si*RPGR^ex1-19^*) were GACGCAGGATACAGCTCTT and TTGTGAGTACAATGAA, respectively. The siRNA duplexes targeting nonspecific sequences were defined as the negative control (NC). The concentration of si*RPGR^ORF15^* and si*RPGR^ex1-19^* siRNA is both 100 nm/L. Cells were transfected with NC, si*RPGR^ORF15^*, or si*RPGR^ex1-19^* by Lipofectamine 3000 Transfection Reagent (L3000015, Invitrogen) for 48 hours or 72 hours and then collected for subsequent experiments.

### Western Blot

Fresh zebrafish eyeballs and cells were collected and lysed in RIPA lysis buffer for protein extraction. Loading buffer was added and mixed with lysates. Then, the mixture was boiled for 5 to 10 minutes, cooled on ice for 5 minutes, and stored at −20°C. Western blotting was performed as described in a previous study.^35^ Protein samples were separated by SDS-PAGE and transferred to nitrocellulose membranes. Next, the membranes were blocked in 5% skim milk for 1 hour at RT and incubated with the dilute solution of primary antibodies overnight at 4°C. The membranes were washed with TBST (20 mM Tris–HCl, 150 mM NaCl 0.05% Tween 20, pH 7.6) 3 times for 5 minutes each and incubated with the HRP-conjugated secondary antibody (1:20,000; Thermo Fisher Scientific) for 2 hours at RT. The protein signals were examined with the SuperSignal Sensitivity Substrate (A38554, Thermo Fisher) using a ChemiDoc XRS+ system (Bio-Rad Life Science, Hercules, CA, USA) and quantified by Quantity One software (Bio-Rad Life Science). The primary antibodies used in this experiment are listed in Supplementary Table S1.

### Phagocytosis and Degradation Assays

The prepared porcine retina photoreceptor OS labeled with FITC (FITC-OS; 46950, Sigma) were chosen to perform phagocytosis assays. RPE-1 and ARPE-19 cells were seeded on coverslips in 12-well plates and transfected with NC and *RPGR^ORF15^* siRNA. After 72 hours, the cells were treated with FITC-OS for 4 hours, and then washed with PBS 5 times to clear unphagocytic FITC-OS. Then, the cells were incubated until 18 and 24 hours, washed with PBS, and fixed with 4% PFA for 15 minutes at RT. The fluorescence pictures were acquired using a confocal laser-scanning microscope (FV3000, Olympus).

### RNA-Seq and Bioinformatics Analysis

Eyeballs from 2 mpf WT and *rpgra^−/−^* zebrafish were dissected, and the RPE layer was collected. Total RNA samples were extracted with Trizol Reagent. RNA sequencing was performed on an Illumina HiSeq2000 platform (Gene Denovo Biotechnology Co. Ltd.). All sequencing data were mapped to the reference genomes (the zebrafish GRCz11 genome) through HISAT2 software. Differentially expressed (DE) genes were determined by the R package DESeq2 using the following cutoff values: fold change > 2 and adjusted *P* value < 0.05. The Kyoto Encyclopedia of Genes and Genomes (KEGG) enrichment analysis was performed on DE genes with DAVID.

### Statistical Analysis

All experiments were performed at least three times independently on three different days, and every time at least three parallel samples were used. Normality was assessed using the D’Agostino-Pearson normality test. Data sets passed normality before parametric tests were performed. For statistical analysis, parametric unpaired *t* test and nonparametric unpaired Mann-Whitney *U* test were used. For extremely small sample sizes (*n* ≤ 5), Student's *t*‐test was performed as recommended.^36^^,^^37^ Results are presented as mean ± SD. Statistical analyses used GraphPad Prism 7, with significance denoted as *P < 0.05, **P < 0.01, ***P < 0.001, and not significant (ns) *P* > 0.05.

## Results

### Rpgra Is Localized in RPE Cells and its Loss Leads to Progressive RPE Degeneration Preceding Photoreceptor Degeneration

**Figure 1.**
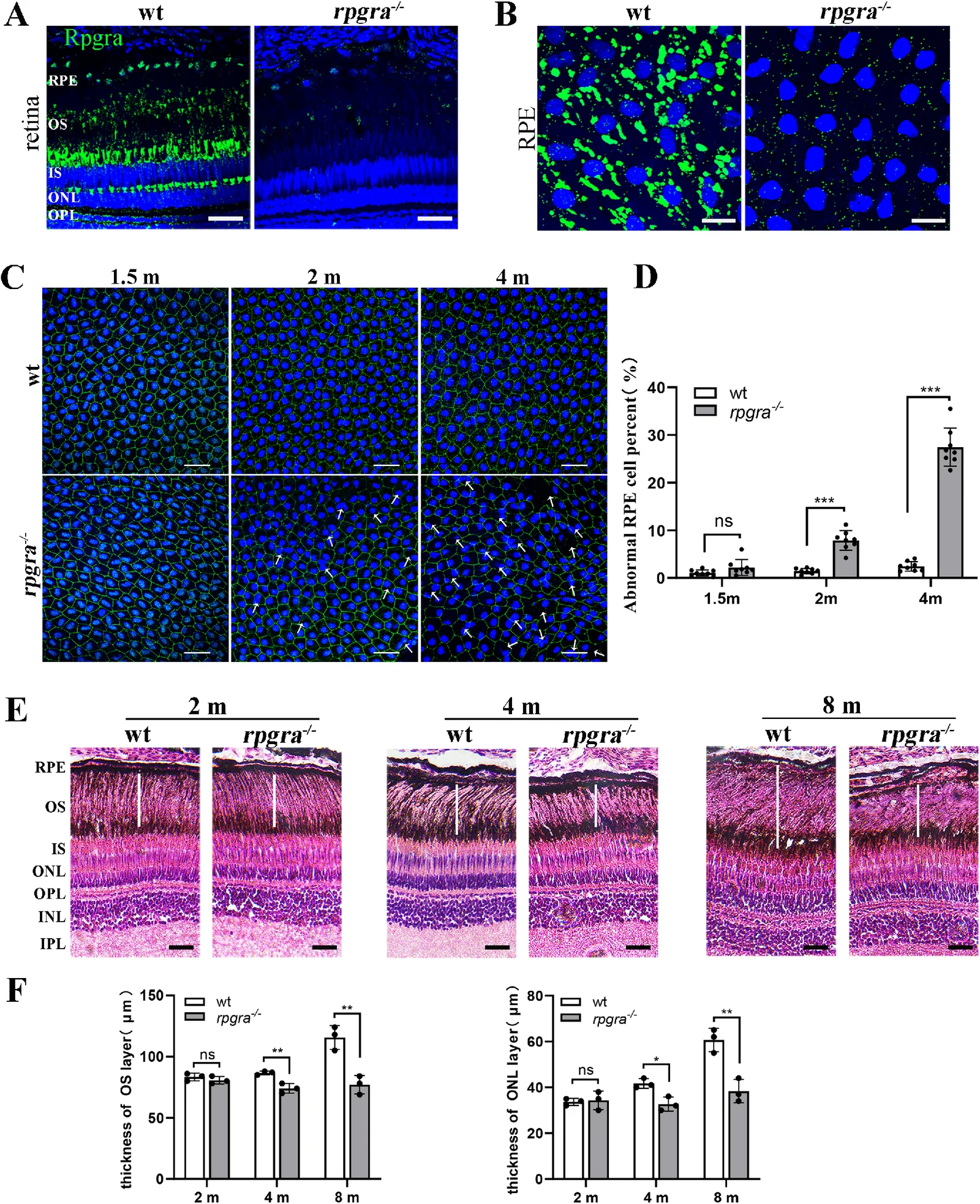
RPE degeneration precedes photoreceptor degeneration in *rpgra^−/−^* zebrafish. (**A,** **B**) Immunostaining of the retinas (**A**) and flat mounted eyecups (**B**) from 2 mpf zebrafish using an antibody against Rpgra (*green*). *Scale bar* = 30 µm. (**C**) Immunofluorescence analysis of flat-mounted eyecups from 1.5, 2, and 4 mpf zebrafish using the anti-ZO-1 antibody (*green*). We defined cells exhibiting loss of polygonal cellular architecture and junctional integrity, or those with irregular nuclear morphology, as abnormal RPE cells. The proportion of RPE cells with abnormal morphology was quantified by manual counting. *White arrows* indicate the aberrant RPE cells. *Scale bar* = 30 µm. (**D**) The number of abnormal RPE cells were quantized for each of the images (*n* = 8). (**E**) Hematoxylin and eosin analysis of cryosections in WT and *rpgra^−/−^* zebrafish at 2, 4, and 8 mpf. *White lines* indicate the length of photoreceptor outer segments. *Scale bar* = 30 µm. INL, inner nuclear layer; IPL, inner plexiform layer; IS, inner segment; ONL, outer nuclear layer; OPL, outer plexiform layer; OS, outer segment; RPE, retinal pigment epithelium. (**F**) The quantitative results of the thickness of OS layer and ONL layer. Three parallel samples were tested for each group (*n* = 3 biological replicates). Data were indicated as mean ± SD. *P < 0.05, **P < 0.01, ***P < 0.001.

We examined the expression of *rpgra* in zebrafish retina using the wild-type (WT) zebrafish and *rpgra* knockout zebrafish line (Supplementary Figs. S2A, S2B). Surprisingly, Rpgra was found to be expressed in RPE cells (Fig. 1A). The result was confirmed by flat mount immunofluorescence analysis (Fig. 1B), suggesting a potential role for *rpgra* in RPE cells.

The morphological characteristics of RPE cells were assessed with zonula occludens-1 (ZO-1) staining by flat mount in WT and *rpgra^−/−^* zebrafish from 1.5 to 4 mpf (Fig. 1C). Quantitative morphometry revealed age-dependent pathological changes in mutants: disruption of polygonal cellular architecture and loss of junctional integrity in RPE cells became detectable at 2 mpf (see Fig. 1C), with a significant increase in the proportion of degenerative RPE cells by 4 mpf (Fig. 1D). These findings imply progressive RPE atrophy due to the loss of *rpgra*.

Histological examination of zebrafish was conducted to investigate retinal phenotypes at the age of 2, 4, and 8 mpf. At 2 mpf, we observed no significant differences in the retinal structure or the thickness of retinal layers between WT and *rpgra*^−/−^ zebrafish (Figs. 1E, 1F). The significant OS and outer nuclear layer (ONL) thinning emerged at 4 mpf and became more pronounced at 8 mpf in *rpgra*^−/−^ zebrafish (see Figs. 1E, 1F). These results indicated that progressive RPE degeneration occurs prior to photoreceptor degeneration in *rpgra*^−/−^ zebrafish, suggesting a crucial role for *rpgra* in maintaining RPE cell homeostasis.

### Loss of *Rpgra* Results in Excessive Lipid Droplet Accumulation in RPE Cells

**Figure 2.**
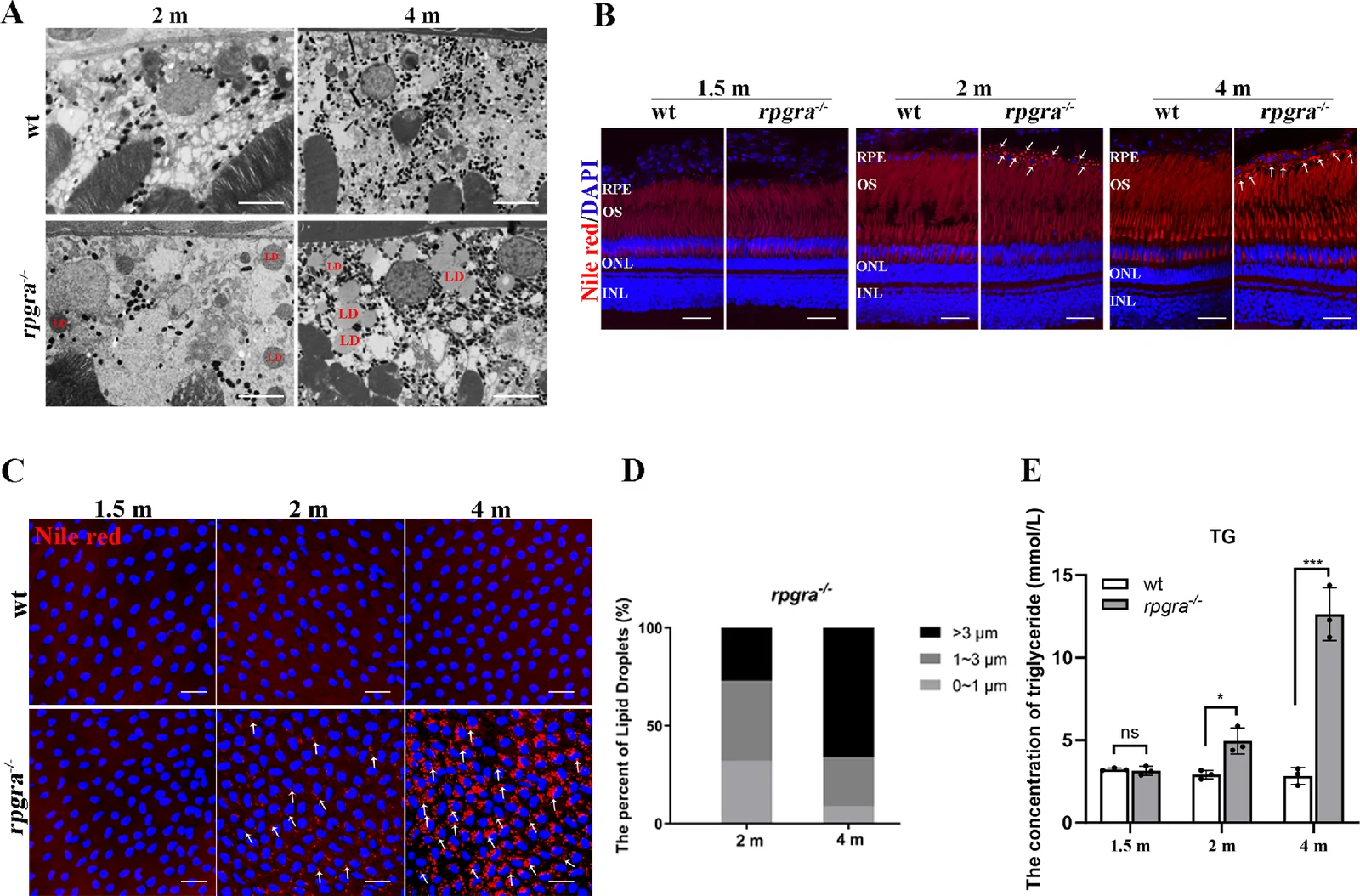
There is lipid accumulation in RPE cells of *rpgra^−/−^* zebrafish. (**A**) Ultrastructural analysis of the retinal RPE layer from 2 and 4 mpf zebrafish. *Scale bar* = 5 µm. (**B**) Nile red staining in frozen retinal sections of 1.5, 2, and 4 mpf zebrafish. *Scale bar* = 30 µm. (**C**) Nile red staining of RPE flat mounted eyecups from 1.5, 2, and 4 mpf zebrafish. *White arrows* indicate lipid droplet (LD). *Scale bar* = 20 µm. (**D**) Diameter of LD size was categorized into three groups: black (>3 µm), dark grey (1–3 µm), and grey (0–1 µm). (**E**) TG concentrations were measured in WT and *rpgra^−/−^* zebrafish (*n* = 3 biological replicates). Data are presented as mean ± SD. Statistical significance is indicated as ns (not significant), *P < 0.05, **P < 0.01, ***P < 0.001.

To delineate the mechanistic basis of RPE degeneration, we performed ultrastructural analysis via TEM in zebrafish RPE cells. Remarkably, LD accumulation was observed in *rpgra^−/−^* zebrafish retinas at both 2 mpf and 4 mpf (Fig. 2A). To confirm these findings, we analyzed lipids in the RPE layer by Nile red staining. At 2 mpf, excessive accumulation of LDs was observed exclusively in the RPE layer of both sectioned and flat-mounted RPE samples from *rpgra*^−/−^ zebrafish (Figs. 2B, 2C). Additionally, the number and diameter of LDs within RPE cells increased with age from 2 mpf to 4 mpf (Figs. 2C, 2D), consistent with the TEM results (Fig. 2A). The concentration of TGs was also significantly elevated in *rpgra*^−/−^ zebrafish (Fig. 2E). Cholesterol has been reported to be closely linked with RPE lipid metabolism.^38^ In 2 mpf *rpgra^−/−^* zebrafish, significantly increased FC accumulation was observed exclusively in the RPE layer using Filipin staining, whereas no noticeable differences were detected at 1.5 mpf (Supplementary Fig. S3A). By 4 mpf, a significantly increased number of FCs in RPE cells and a marked increased FC content were observed compared with 2 mpf *rpgra*^−/−^ zebrafish (see Supplementary Figs. S3A, S3B). These results suggest that the excessive accumulation of LDs and FCs in RPE cells may be linked to the progression of RPE atrophy and dysfunction, ultimately contributing to photoreceptor degeneration in *rpgra^−/−^* zebrafish.

**Figure 3.**
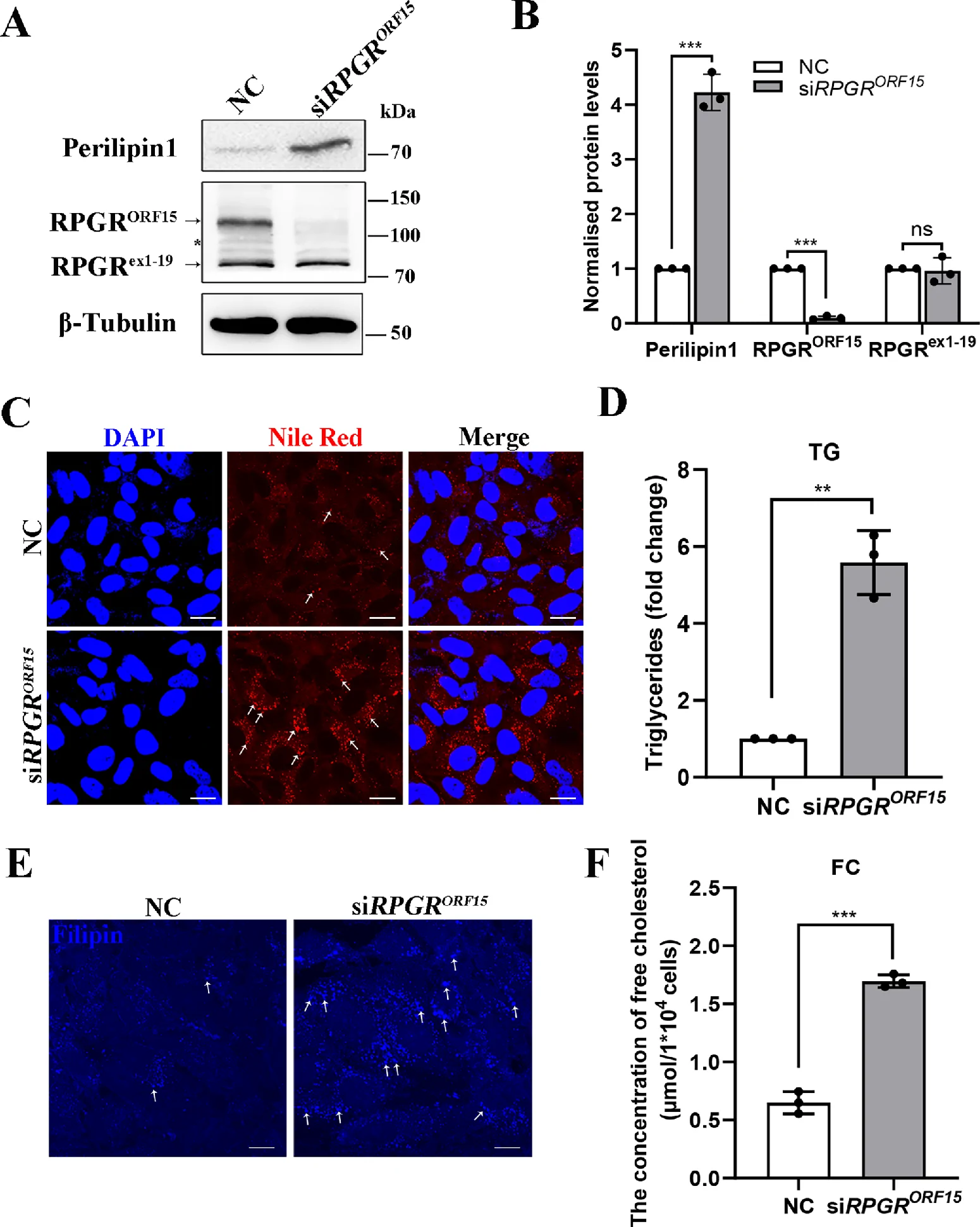
Lipid droplet accumulation in si*RPGR^ORF15^* RPE-1 cells. (**A**) The protein level of Perilipin1, RPGR^ORF15^, and RPGR^ex1-19^ in NC, and si*RPGR^ORF15^* RPE-1 cells were detected and quantified (**B**). The * may indicate an alternatively spliced or post-translationally modified isoform (*n* = 3 biological replicates). (**C**) Nile red staining and TG concentrations (**D**) were measured in both NC and si*RPGR^ORF15^* RPE-1 cells, with *arrows* highlighting the LD. *Scale bar* = 20 µm (*n* = 3 biological replicates). (**E**) Filipin staining and FC concentrations (**F**) were measured in NC and si*RPGR^ORF15^* RPE-1 cells. The *white arrows* mark the FC. *Scale bar* = 10 µm (*n* = 3 biological replicates). Statistical significance is indicated as not significant (ns), **P < 0.01, ***P < 0.001.

To confirm the above results in human RPE cells, we used siRNA to knock down *RPGR^ORF15^* specifically in RPE-1 and ARPE-19 cells in vitro. The protein level of *RPGR^ORF15^* was significantly decreased in these cells (Figs. 3A, 3B; Supplementary Figs. S3C, S3D). The protein level of *RPGR^ex1-19^* remained unchanged compared with the NC group, confirming the specificity of the *RPGR^ORF15^* knockdown. Consistently, the upregulation of the LD marker protein Perilipin1, along with LD accumulation, FC accumulation, and increased concentrations of TG and FC was observed in *RPGR^ORF15^*-knockdown RPE-1 and ARPE-19 cells (see Figs. 3A–F; Supplementary Figs. S3C–S3H). These results demonstrated that *RPGR^ORF15^* is a critical regulator of lipid homeostasis in RPE cells.

To determine whether the LDs accumulation in *rpgra*^−/−^ zebrafish and si*RPGR^ORF15^* RPE-1 cells is specifically dependent on the ORF15 domain, we transfected siRNA targeting *RPGR^ex1-19^* into RPE-1 cells to clarify the relationship between *RPGR^ex1-19^* and LDs’ accumulation. Compared with the NC group, downregulation of *RPGR^ex1-19^* did not result in LDs or FC accumulation in the *RPGR^ex1-19^* knockdown RPE-1 cells (Supplementary Figs. S4A–S4E). These results indicated that excessive lipid accumulation in RPE cells is a consequence of the loss of RPGR^ORF15^ rather than RPGR^ex1-19^, indicating a specific causal relationship between *RPGR^ORF15^* and lipid metabolism disorders.

### *RPGR^ORF15^* Deficiency Impairs Lysosome in Zebrafish and Human RPE Cells

**Figure 4.**
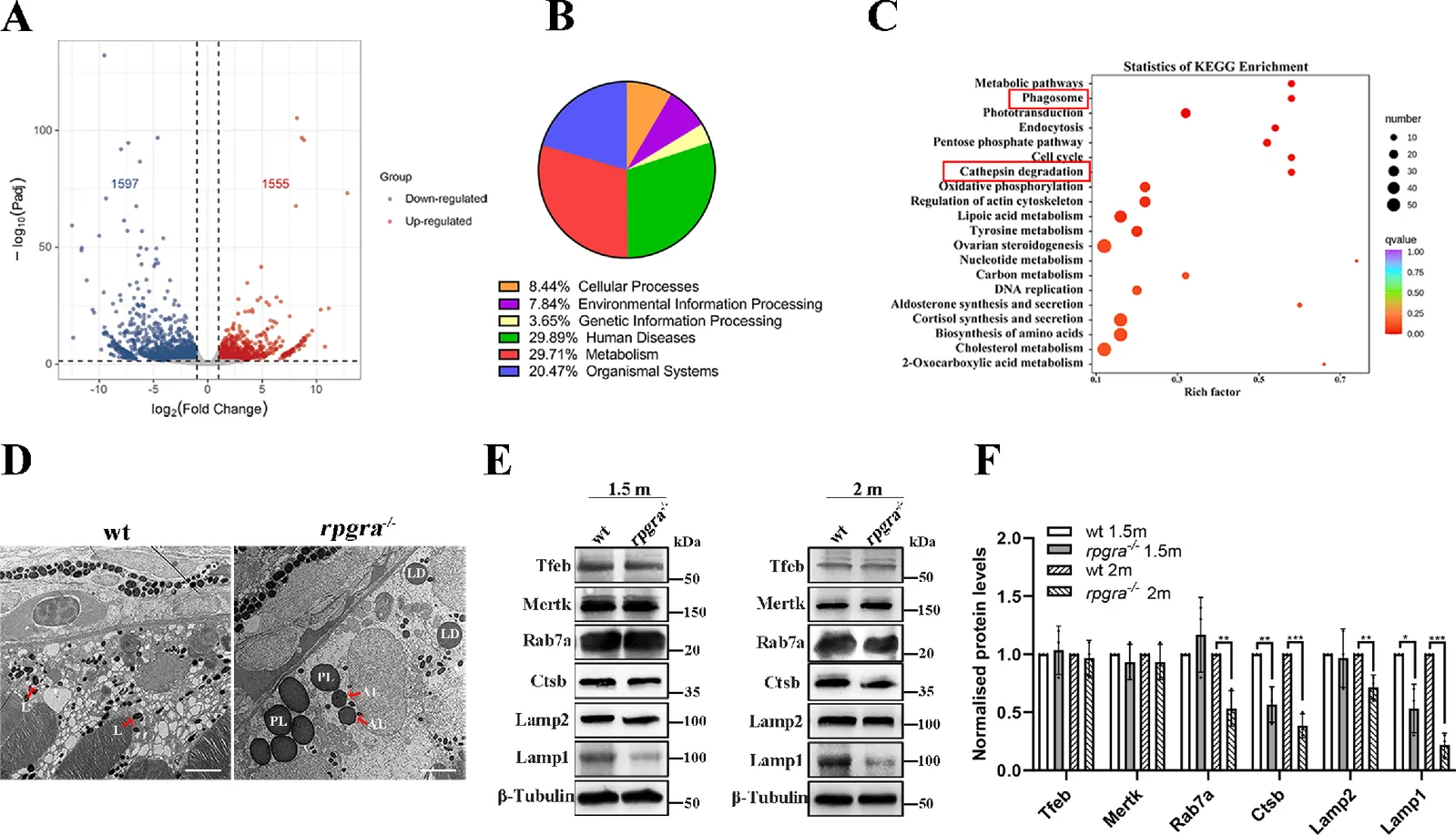
Loss of *rpgra* alters phagocytosis-related pathways and protein levels in zebrafish RPE cells. (**A**) Volcano plot depicting differentially expressed genes in *rpgra^−/−^* zebrafish compared with WT. (**B**) The differentially expressed genes from top 50 KEGG terms were classified into the 6-enrichment categories. (**C**) KEGG pathways enrichment analysis based on differentially expressed genes. (**D**) TEM images of retinal ultrathin sections from WT and *rpgra^−/−^* zebrafish at 2 mpf. AL, abnormal lysosomes; PL, undegraded phagolysosomes; L, lysosomes. *Scale bar* = 3 µm. (**E**) Detection and quantification (**F**) of the protein level of phagocytosis related-gene in zebrafish RPE cells by Western blot at 1.5 mpf and 2 mpf (*n* = 3 biological replicates). Data are presented as mean ± SD. Statistical significance is indicated as not significant (ns), *P < 0.05, **P < 0.01, ***P < 0.001.

To investigate the underlying mechanism of lipid accumulation in *rpgra^−/−^* zebrafish and si*RPGR*^ORF15^ RPE cells, transcriptomic profiling of RPE tissues from WT and *rpgra^−/−^* zebrafish at 2 mpf was performed by RNA sequencing (RNA-seq). A total of 3152 differentially expressed genes were identified in *rpgra*^−/−^ zebrafish RPE cells, with 1555 upregulated and 1597 downregulated (Fig. 4A). The KEGG pathway analysis revealed that metabolic processes account for 29% of the regulated pathways (Fig. 4B). Additionally, phagosome and cathepsin degradation pathways were enriched (Fig. 4C). Consistently, excessive LDs, abnormal lysosomes (ALs) and undegraded phagolysosomes (PLs) were observed in the RPE layer of 2 mpf *rpgra*^−/−^ zebrafish using TEM (Fig. 4D), suggesting compromised lysosomal clearance capacity.

**Figure 5.**
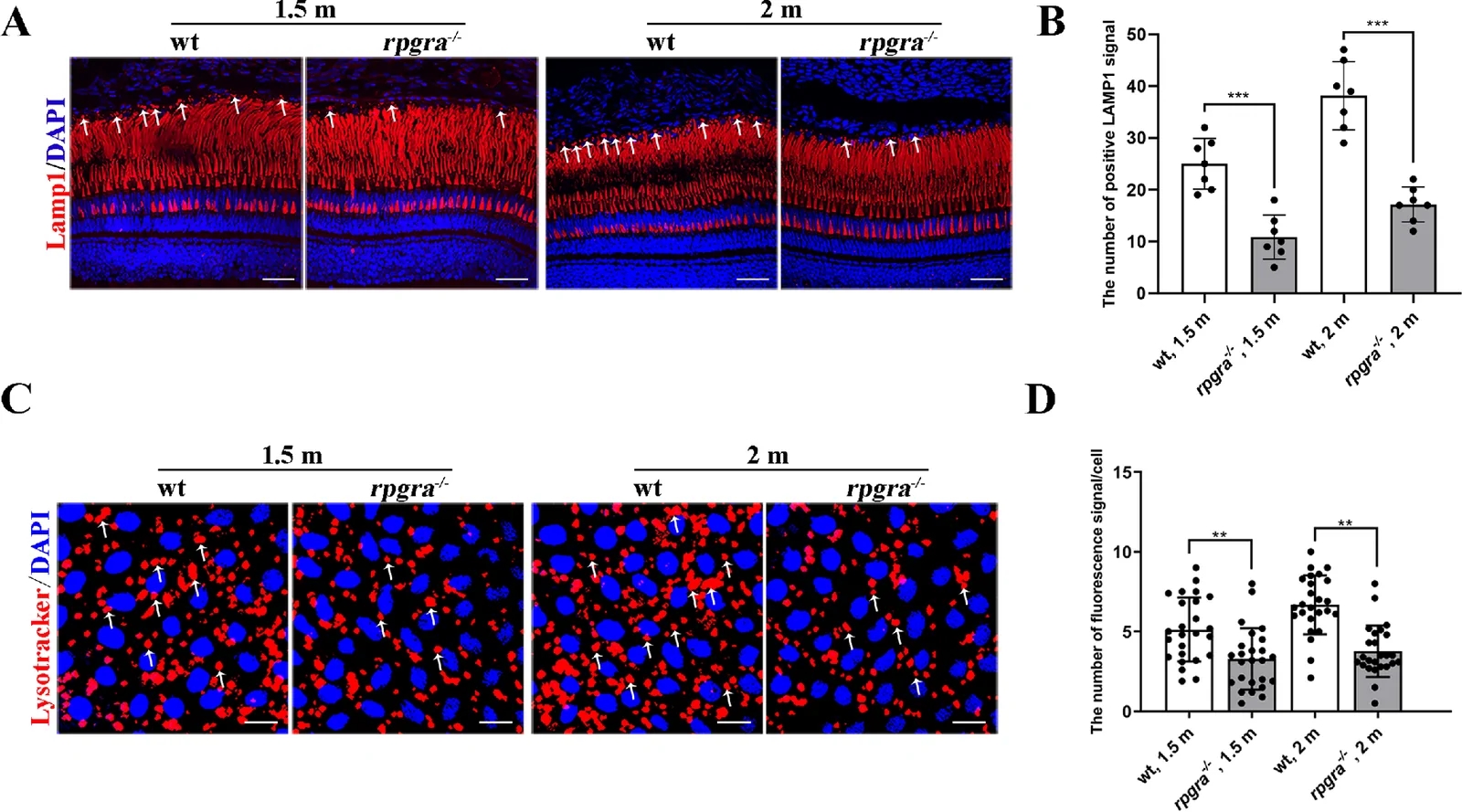
The deficiency of *rpgra* results in impaired lysosome in zebrafish retina. (**A**) Immunostaining analysis and quantification (**B**) for LAMP1 on retinal cryosection of 1.5 and 2 mpf zebrafish. Signals are indicated by *white arrows* (*n* = 7). *Scale bar* = 30 µm. (**C**) Immunofluorescence staining and quantification (**D**) for Lysotracker on RPE flat-mounted eyecups of 1.5 and 2 mpf zebrafish. Signals are represented by *white arrows* (*n* = 25). *Scale bar* = 10 µm. Data are presented as mean ± SD. Statistical significance is indicated as not significant (ns), *P < 0.05, **P < 0.01, ***P < 0.001.

To determine whether RPE phagocytosis and lysosomal dysfunction occur in *rpgra^−/−^* zebrafish, we examined the protein levels of phagocytosis-associated genes (involved in engulfment and degradation) in zebrafish. A significant decrease in LAMP1 (a marker for lysosome biogenesis) and CTSB was first detected in 1.5 mpf *rpgra^−/−^* zebrafish (Figs. 4E, 4F). Subsequently, downregulation of LAMP2 and Rab7a (markers for late endosomes) was observed in 2 mpf *rpgra^−/−^* zebrafish. Immunofluorescence analysis of LAMP1 (Figs. 5A, 5B) and lysotracker (a lysosomes marker) staining (Figs. 5C, 5D) corroborated the Western blot results, both indicating lysosomal dysfunction in *rpgra^−/−^* zebrafish.

**Figure 6.**
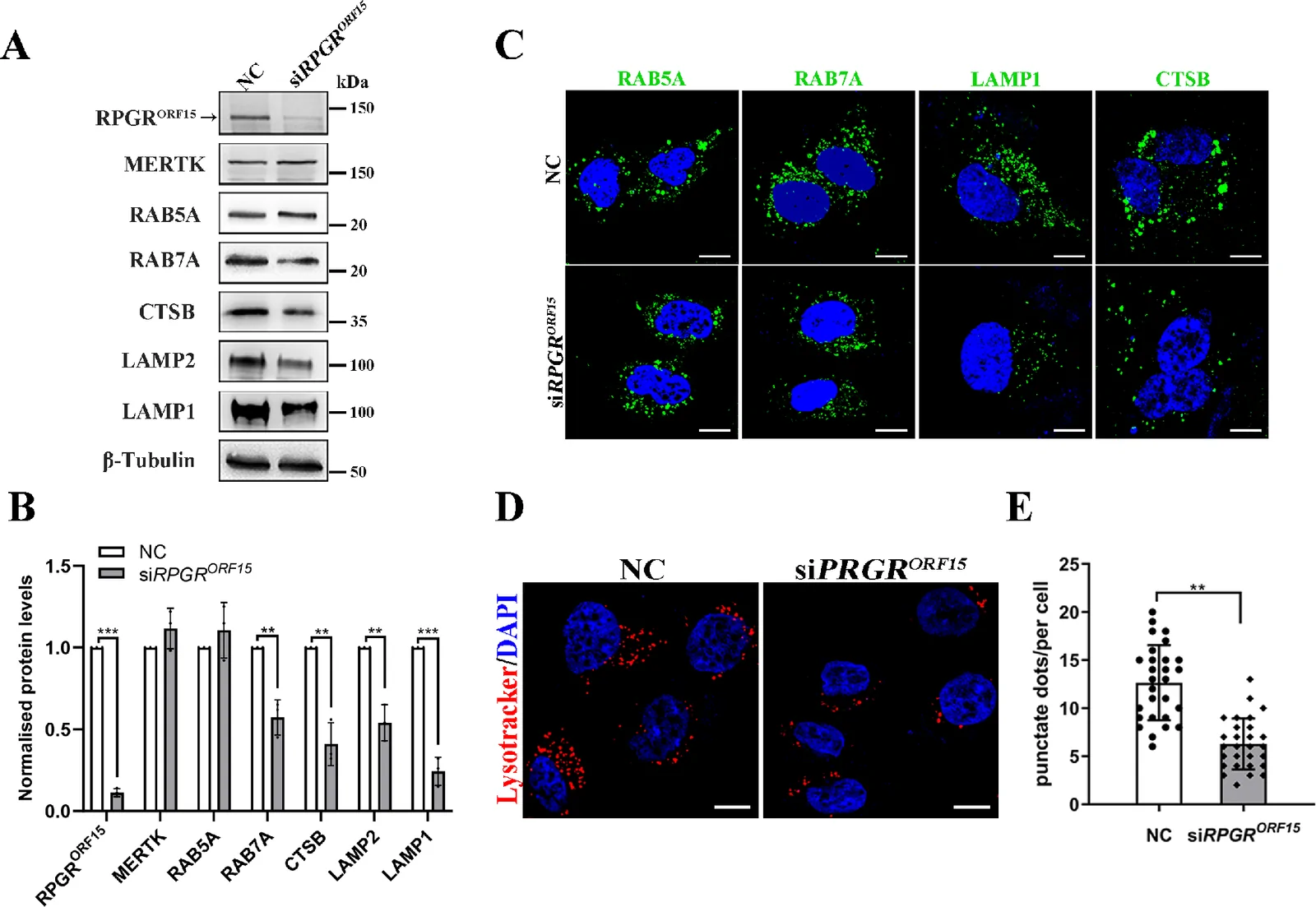
The deficiency of RPGR^ORF15^ leads to lysosome dysfunction in RPE-1 cells. (**A,** **B**) Protein levels and quantification of phagocytosis related-genes in NC and si*RPGR^ORF15^* RPE-1 cells (*n* = 3 biological replicates). (**C**) Immunostaining of early endosomes (Rab5a), Rab7a, LAMP1, and CTSB in NC and si*RPGR^ORF15^* RPE-1 cells. *Scale bar* = 10 µm. (**D,** **E**) Lysotracker staining and quantification of signal dots per cell in NC and si*RPGR^ORF15^* RPE-1 cells (*n* = 28). *Scale bar* = 10 µm. Data are presented as mean ± SD. Statistical significance is indicated as not significant (ns), *P < 0.05, **P < 0.01, ***P < 0.001.

Decreased expression of LAMP1 and CTSB was further confirmed by Western blot (Figs. 6A, 6B; Supplementary Figs. S5A, S5B), immunofluorescent analysis (Fig. 6C; Supplementary Fig. S5C), and lysotracker staining (Figs. 6D, 6E; Supplementary Figs. S5D, S5E) in both si*RPGR^ORF15^* RPE-1 and si*RPGR^ORF15^* ARPE-19 cells. These results suggest that impaired lysosome may be the key inducers of excessive lipid accumulation in *rpgra*^−/−^ zebrafish.

To investigate whether lysosomal dysfunction is responsible for the lipid disturbance due to RPGR^ORF15^ loss, we performed rescue experiments by overexpression of LAMP1 (LAMP1-GFP) in ARPE-19 cell lines. The result of Nile red staining showed that LAMP1 overexpression significantly reduced the LDs accumulation in si*RPGR^ORF15^* ARPE-19 cells (Supplementary Figs. S8A, S8B). We further measured intracellular the TG levels using a triglyceride kit. The levels of TG were partially restored by LAMP1 overexpression, consistent with Nile red staining results (Supplementary Fig. S8C). These results suggest that LAMP1 functions as a critical downstream target of RPGR^ORF15^ and lysosomal abnormality is a major contributor to LDs accumulation in si*RPGR^ORF15^* ARPE-19 cells.

Transcription factor EB (TFEB) is a master regulator of lysosome biogenesis.^39^ Western blot results showed that the TFEB protein level remained unchanged (see Figs. 4E, 4F). Because nuclear translocation of TFEB is essential for the transcription of lysosome related genes, we further examined whether RPGR^ORF15^ deficiency could inhibit this process. Then, we analyzed the localization of TFEB protein by immunofluorescence. The results showed that there was no significant change in the nuclear translocation of TFEB between groups in the NC and si*RPGR^ORF15^* RPE-1 cells (Supplementary Figs. S6A, S6B).

### RPGR^ORF15^ Deficiency Affects the Degradation Capability of RPE Due to Lysosomal Dysfunction

**Figure 7.**
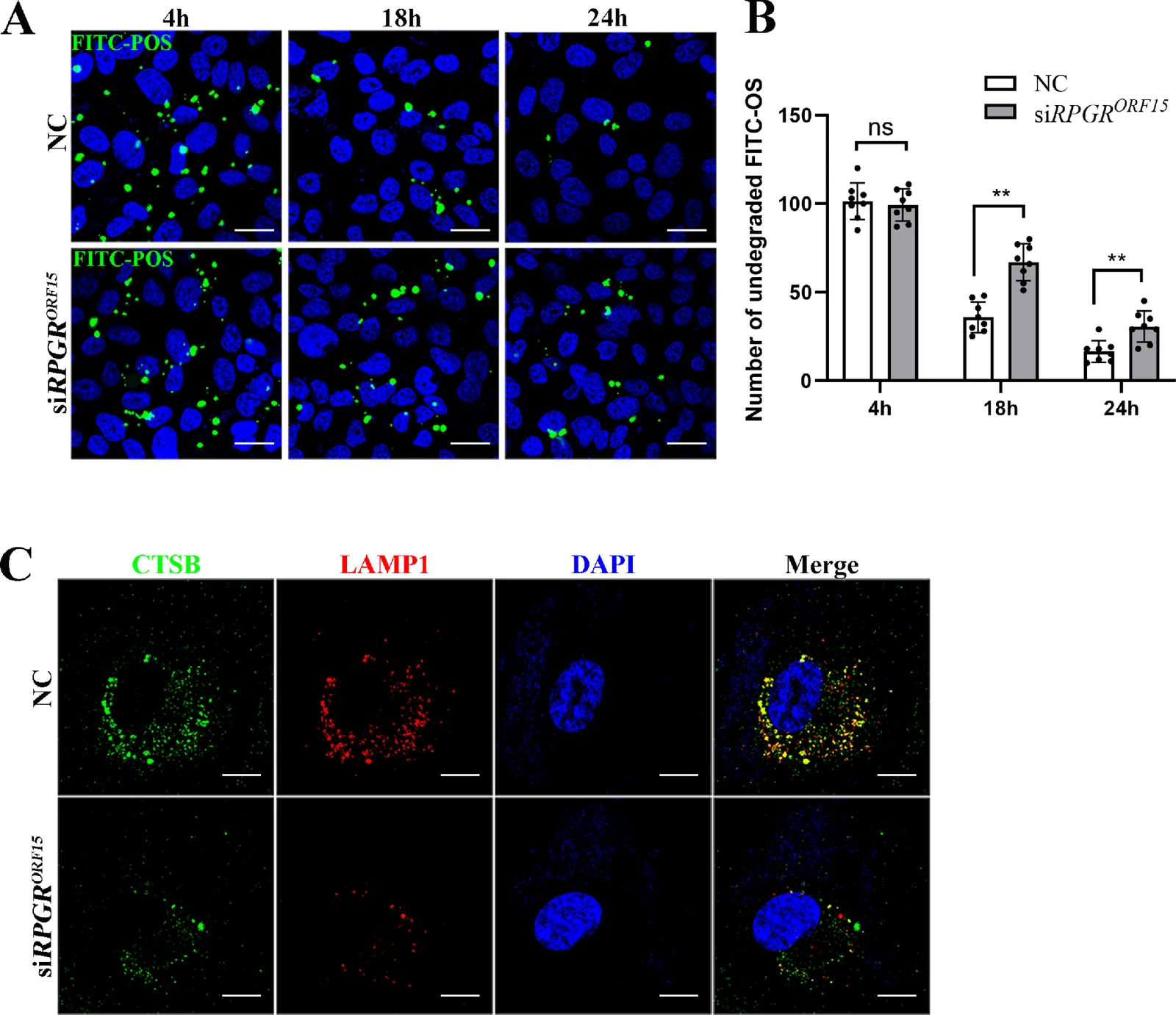
Knockdown of *RPGR^ORF15^* resulted in lysosomal degradation dysfunction rather than early phagocytosis in RPE-1 cells. (**A**) The phagocytosis and degradation capacity of NC and si*RPGR^ORF15^* RPE-1 cells and (**B**) quantification of FITC-OS (*n* = 8). *Scale bar* = 20 µm. (**C**) Reduced colocalization between LAMP1 and CTSB in si*RPGR^ORF15^* RPE-1 cells. *Scale bar* = 20 µm. Data are presented as mean ± SD. Statistical significance is indicated as not significant (ns), *P < 0.05, **P < 0.01.

Next, we tested whether the phagocytosis was impaired in si*RPGR^ORF15^* RPE-1 and ARPE-19 cells. During the phagocytosis of photoreceptor OS, RPE cells bind and internalize OS within 2 hours, engulf them in 2 to 6 hours, and digest the engulfed OS after 16 hours.^35^ To determine the specific stage of RPE phagocytosis dysfunction in *rpgra^−/−^* zebrafish, we used FITC-labeled OS (FITC-OS) to assess the engulfment and degradation capabilities of NC and si*RPGR^ORF15^* RPE cells. After incubating si*RPGR^ORF15^* or the NC group, RPE-1 or ARPE-19 cells with FITC-OS (Figs. 7A, 7B; Supplementary Figs. S7A, S7B) for 4 hours, the results showed that si*RPGR^ORF15^* RPE-1 and ARPE-19 cells have comparable engulfment efficiency to controls, indicating that *RPGR^ORF15^* knockdown does not affect the early steps of RPE phagocytosis. This is consistent with the unchanged level of Mertk protein (a marker for engulfment) in *rpgra^−/−^* zebrafish and si*RPGR^ORF15^* RPE-1 and ARPE-19 cells (see Figs. 4E, 4F, 6A, 6B; Supplementary Figs. S5A, S5B).

Striking divergence emerged during substrate degradation. After 18 hours and 24 hours, *RPGR^ORF15^* knockdown suppressed the degradation capability of RPE cells for FITC-OS (Figs. 7A, 7B; Supplementary Figs. S7A; S7B). This observation aligns with the decreased colocalization between LAMP1 and CTSB, which is key to degrade substances within lysosomes (Fig. 7C; Supplementary Fig. S7C). Taken together, these results suggest that knockdown of *RPGR^ORF15^* impairs the degradation capability of RPE cells due to lysosomal dysfunction.

## Discussion

Although many reports show RPGR is localized within photoreceptors and involved in intracellular transport,^13^^,^^30^^,^^40^ its role in RPE cells remains unexplored. Several major new findings of this study include the following: first, we discovered that *RPGR^ORF15^* is expressed in RPE cells, and deficiency of *RPGR^ORF15^* results in RPE degeneration. Second, *RPGR^ORF15^* plays an essential role in the lipid metabolism process, which may be one of the leading causes of *RPGR^ORF15^* associated RP. Third, RPGR^ORF15^ deficiency impairs lysosomal formation, which leads to lipid metabolism disorder and rapid RPE atrophy, followed by retina degeneration.

Currently, the role of RPGR in RPE cells remains unclear. Previous studies have reported that RPGR is primarily expressed in photoreceptor cells.^29^^,^^30^^,^^41^ Compared with previous reports, we found that RPGR is expressed not only in photoreceptor cells, but also in RPE cells of zebrafish. To investigate whether our findings are zebrafish-specific, the conservation between zebrafish and human RPE cell lines has been confirmed (see Fig. 3A; Supplementary Fig. S3C). Here, we found that *RPGR^ORF15^* knockdown impaired lysosomal degradation in RPE phagocytosis and uncovered an indispensable role of RPGR for regulating lysosome biogenesis and function in RPE cells and its impact on photoreceptor degeneration. These results suggest that *RPGR^ORF15^* may play a crucial role in lysosome biogenesis and function. However, our results showed that *RPGR^ORF15^* deficiency neither altered the protein level of TFEB nor affected its nuclear translocation. Compared with previous studies demonstrating that TFEB regulates lysosome biogenesis,^42^^,^^43^ our findings suggest that *RPGR^ORF15^* modulates lysosomes in a TFEB-independent manner. It is possible that *RPGR^ORF15^* regulates LAMP1 directly or through alternative pathways, such as ZKSCAN3^44^ and MITF.^45^ The exact mechanisms will be further explored in subsequent studies.

In addition to the degeneration of photoreceptor cells, patients with RPGR mutations exhibit various degrees of RPE atrophy.^3^^,^^15^^,^^46^ The existing literature indicates that RPGR mutations leads to photoreceptor degeneration by ciliary dysfunction and disrupting cargo transport.^12^^,^^14^^,^^47^^,^^48^ A recent study demonstrated that RPGR functions as a guanine nucleotide exchange factor, interacting with RAB37 to promote autophagy, suggesting that photoreceptor degeneration in Rpgr knockout mice is caused by the impaired autophagy.^41^ However, the pathological mechanisms underlying RPE atrophy in patients with RPGR mutations have not yet been reported. This study provides a possible mechanistic explanation for RPE atrophy and retinal degeneration in patients with RPGR mutations. We demonstrate that *RPGR^ORF15^* depletion disrupts lysosomal function (see Figs. 4E, 4F, 5A–D) and phagolysosomal degradation (see Figs. 7A–C), creating a “lipid clearance crisis” that resulted in RPE degeneration (see Fig. 1C). Deficiency of another RP-associated gene, *CYP4V2*, results in RPE lipid accumulation, which precedes RPE atrophy and photoreceptor degeneration.^25^ In *Ambra1^+/gt^* mice, alterations in proteostasis within RPE result in RPE atrophy, increased lipid peroxidation, metabolic dysfunction, and lipofuscin formation later, and a heightened susceptibility to aged-associated retinal degeneration.^49^ Our results were consistent with previous reports.^25^^,^^49^ These findings suggest that the structural abnormalities of the RPE and lipid accumulation resulting from *RPGR^ORF15^* mutations, or other genes, may be key drivers of retinal degeneration.

In *RPGR^ORF15^* mutant mice, marked weakening of RPE tight junctions compromises the integrity of the outer blood-retina barrier.^50^ However, the reasons for this weakening and the relationship between RPE cell dysfunction and other retinal layers remain unaddressed. Impairments in lysosome within RPE cells have been identified as key contributors to retinal degenerative diseases, such as RP and AMD.^51^^,^^52^ Notomi et al. demonstrated that dysregulated phagocytic degradation of photoreceptor OS in RPE cells leads to accumulation of basolateral deposits.^22^ Lysosomal impairments in conjunction with lipofuscin accumulation are pivotal inducers of RPE cell degeneration.^49^ Yako et al. demonstrated that LDs’ accumulation causes RPE dysfunction, implying its role in the pathogenesis of retinal degenerative diseases.^53^ In *rpgra^−/−^* zebrafish retina, we observed lysosome biogenesis significantly decreased at 1.5 mpf, followed by LD accumulation and RPE degeneration at 2 mpf, and photoreceptor cell degeneration was first observed at 4 mpf. Therefore, we can speculate that lysosomal dysfunction disrupts lipid metabolism, leading to RPE atrophy and subsequently photoreceptor cell degeneration in *rpgra^−/−^* zebrafish.

In conclusion, this study identifies *RPGR^ORF15^* as a critical regulator of lysosomal function and lipid metabolism balance in RPE cells, whose deficiency drives RPE and photoreceptor degeneration. By identifying conserved phagolysosomal degradation defects across zebrafish and human cell lines, we highlight lysosomal reactivation as a therapeutic strategy for RPGR-related retinopathies.

## Supplementary Material

Supplement 1
