# Supplementary material for: RPGRORF15 Mutations Disrupt Lysosomal Lipid Metabolism in Retinal Pigment Epithelium Cells and Cause Retinitis Pigmentosa
Source: Invest Ophthalmol Vis Sci. 2025 Nov 25;66(14):61. doi: 10.1167/iovs.66.14.61 (PMC12663888; doi:10.1167/iovs.66.14.61)
Supplement: Supplement 1 [file iovs-66-14-61_s001.docx]

**Supplementary material**

**
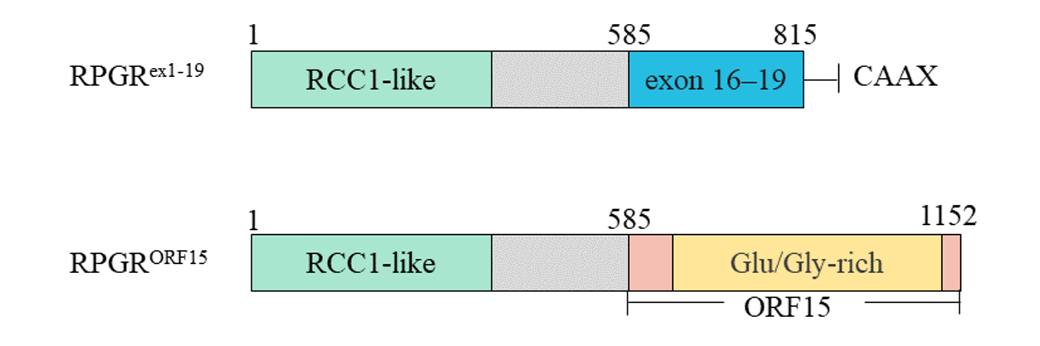
**

Figure S1. Schematic representation of RPGR^ex1-19^ and RPGR^ORF15^ protein isoforms. The N-terminal RCC1-like domain (green) of RPGR is shared between RPGR^ORF15^ and RPGR^ex1–19^ isoforms. But RPGR^ex1-19^ and RPGR^ORF15^ have different C-terminals. The RPGR^ex1-19^ has an isoprenylation site (CAAX) at the C-terminus, whereas RPGR^ORF15^ contains a repetitive glycine and glutamic acid-rich domain (yellow) within RPGR^ORF15^.


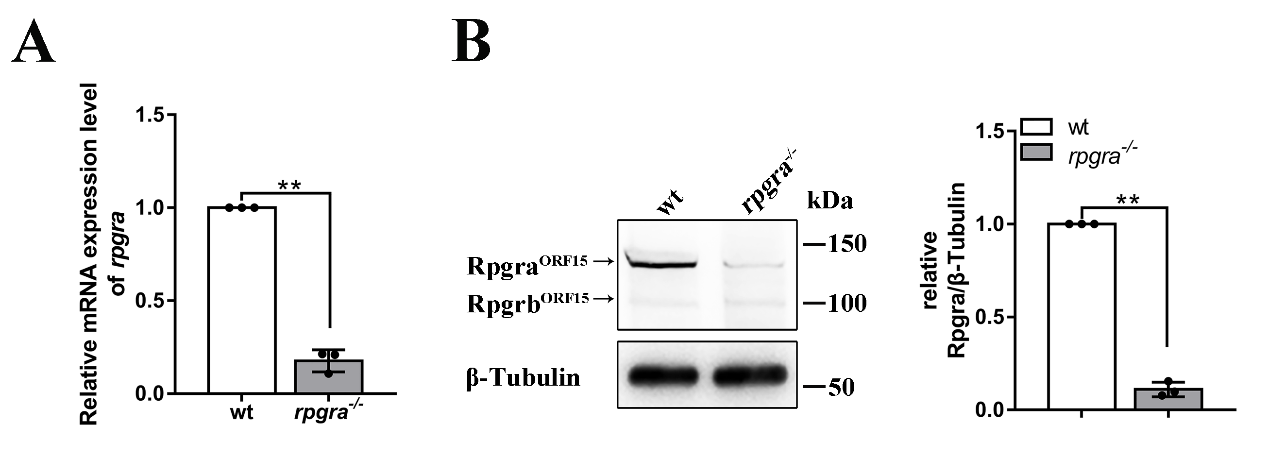


**Figure S2.** The validation of targeted mutagenesis of *rpgra* in zebrafish retina. (A) Relative mRNA expressions of *rpgra* detected by qRT-PCR in 2 mpf WT and *rpgra^-/-^* zebrafish. The β-Tubulin served as endogenous control. *n* = 3 biological replicates. (B) Rpgra protein levels of WT and *rpgra^-/-^* zebrafish, against β-Tubulin expression, at 2 mpf were detected by western blot. The Rpgrb band marked by arrow was almost undetectable and had no significant changes between WT and *rpgra^-/-^* zebrafish. The protein level of Rpgra were quantized. *n* = 3 biological replicates. Data were indicated as mean ± SD. **p* < 0.05, ***p* < 0.01.


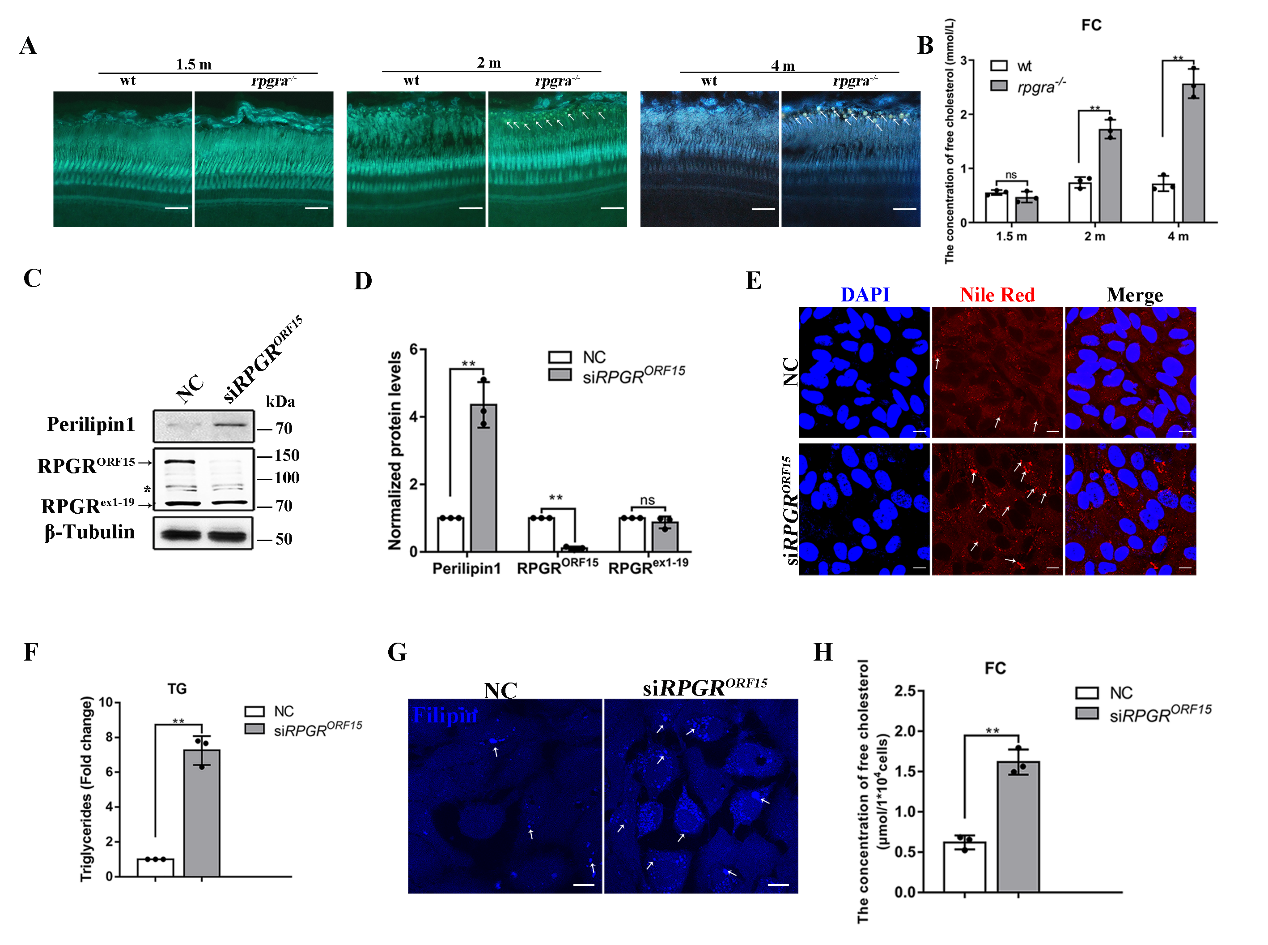


**Figure S3.** FC increased in *rpgra* knockout zebrafish and lipid accumulated in si*RPGR^ORF15^* ARPE-19 cells. (A) Filipin staining of FC in retinal frozen sections of WT and *rpgra* knockout zebrafish at 1.5, 2 and 4 mpf. White arrows represented FC. Scale bar: 30 μm. (B) Quantitative analysis of the concentration of FC. *n* = 3 biological replicates. (C) The protein levels of Perilipin1, RPGR^ORF15^ and RPGR^ex1-19^ in NC and si*RPGR^ORF15^* ARPE-19 cells. The * may represent an alternatively spliced or post-translationally modified isoform. (D) Quantitative analysis of the western blot in(C) *n* = 3 biological replicates. (E)More LDs and FC (G) accumulated in si*RPGR^ORF15^* ARPE-19 cells. White arrows indicated LDs and FC, respectively. Scale bar: 20 μm. (F, H) Measurement of TG and FC concentrations in NC and si*RPGR^ORF15^* ARPE-19 cells. *n* = 3 biological replicates. The results were shown as mean ± SD. ns, not significant, **p*<0.05, ***p*<0.01.


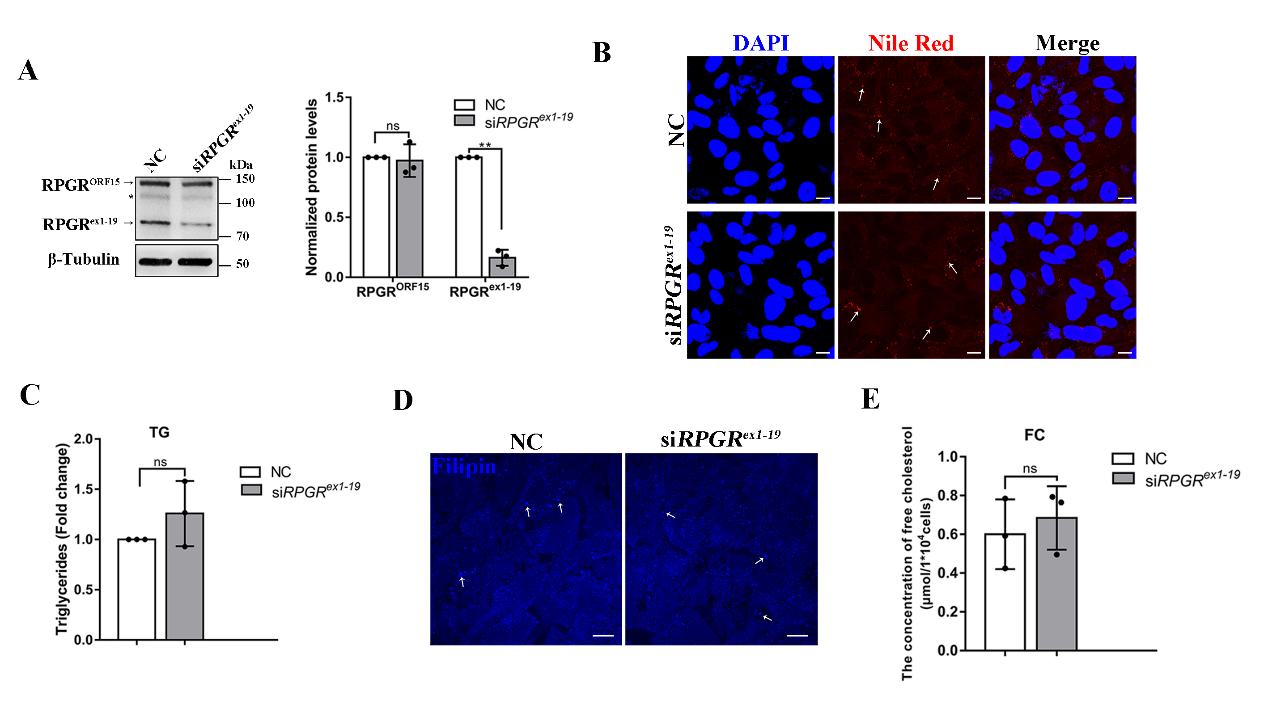


**Figure S4.** There is no lipid accumulation in si*RPGR* *^ex1-19^* RPE-1 cells.

(A) The protein levels of RPGR^ORF15^ and RPGR^ex1-19^ in NC and si*RPGR^ex1-19^* RPE-1 cells. The * may represent an alternatively spliced or post-translationally modified isoform. Quantitative analysis of the western blot. *n* = 3 biological replicates. Compared with NC group, no LDs (B) and free cholesterol (FC) (D) accumulated in si*RPGR^ex1-19^* RPE-1 cells. White arrows indicated LDs and FC, respectively. Scale bar: 20 μm. Measurement of TG (C) and FC (E) concentrations in NC and si*RPGR^ex1-19^* RPE-1 cells, *n* = 3 biological replicates. The results were shown as mean ± SD. ns, not significant, **p*<0.05, ***p*<0.01.


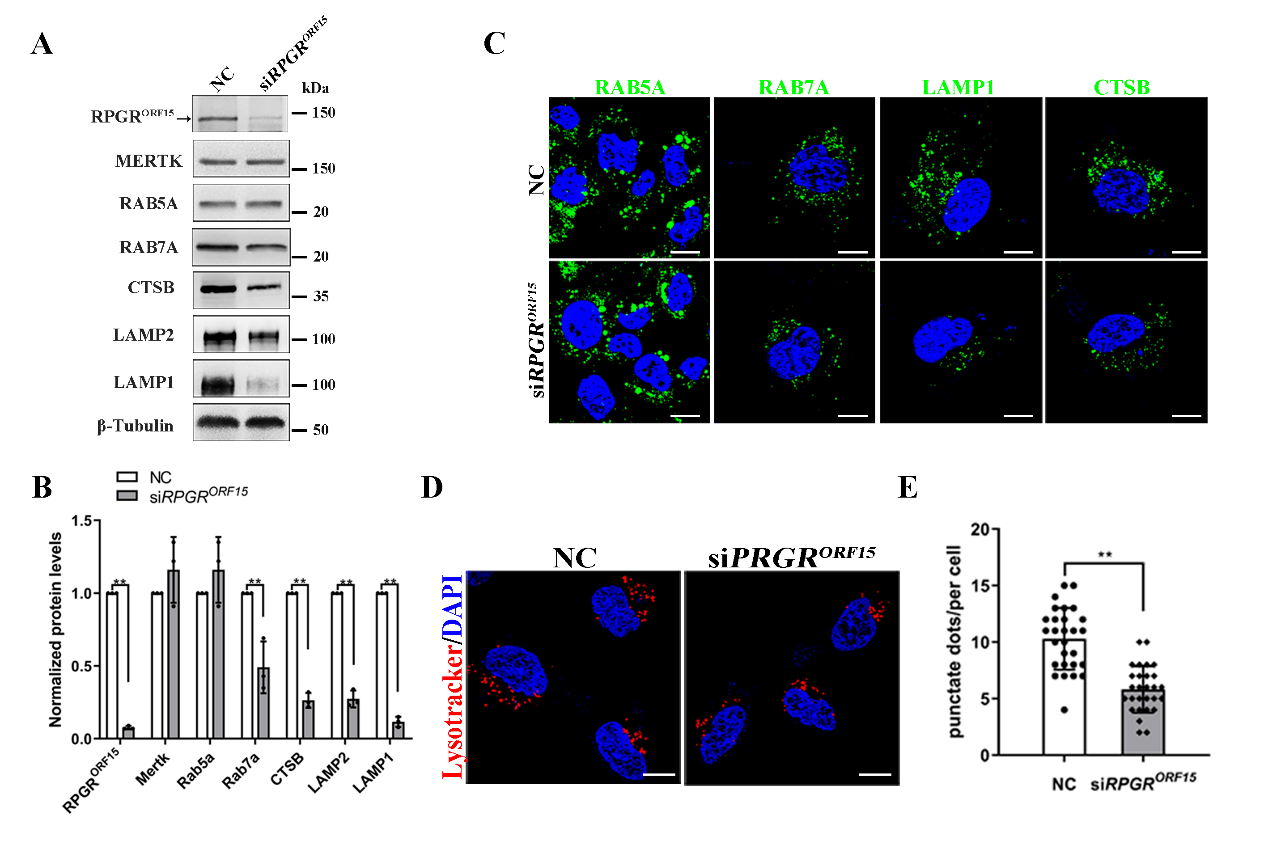


**Figure S5.** Knockdown of *RPGR^ORF15^* resulted in lysosomal dysfunction in ARPE-19 cells. (A) Western blot of Phagosome and Cathepsin degradation related-gene in NC and si*RPGR^ORF15^* ARPE-19 cells. (B) Quantification of the detected protein in (A). *n* = 3 biological replicates. (C) Immunostaining analysis of Rab5a, Rab7a, LAMP1 and CTSB in NC and si*RPGR^ORF15^* ARPE-19 cells. Scale bar: 10 μm. (D) Immunostaining analysis of Lysotracker in NC and si*RPGR^ORF15^* ARPE-19 cells. (E) The punctate dots of each cell were counted. n = 28, scale bar: 10 μm. Data were displayed as mean ± SD. ns, not significant, **p* < 0.05, ***p* < 0.01.


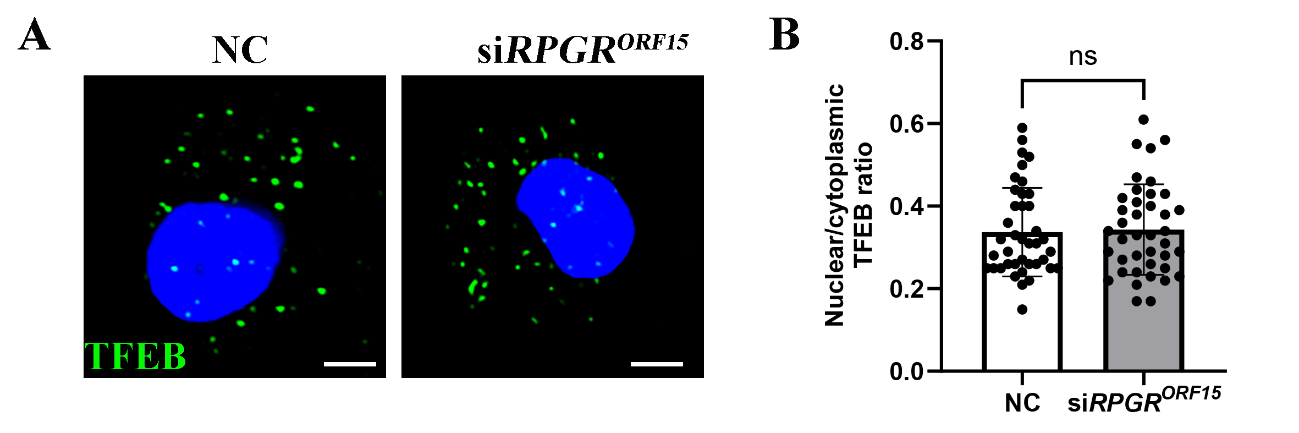


Figure S6. Immunofluorescence of TFEB (green) in NC and si*RPGR^ORF15^* RPE-1 cell. (A) localization of TFEB protein in the nucleus and cytoplasm. (B) Quantification of nuclear/cytoplasmic TFEB ratio in A. n = 40. Scale bar = 5 μm. *n* = 3 biological replicates. ns, not significant.


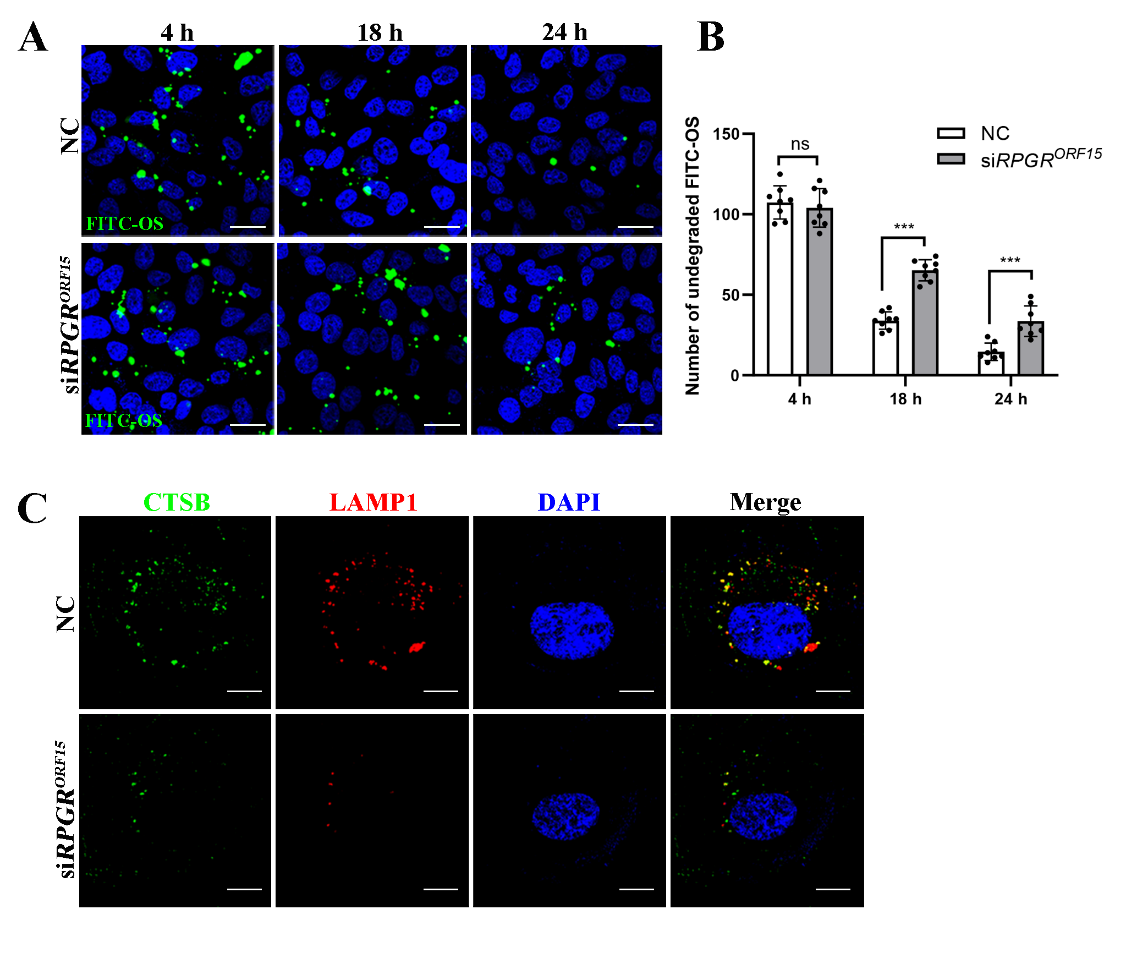


**Figure S7.** Knockdown of *RPGR^ORF15^* resulted in lysosomal degradation dysfunction rather than early phagocytosis in ARPE-19 cells. (A) FITC-OS was used to detect the phagocytosis and degradation function in NC and si*RPGR^ORF15^* ARPE-19 cells. (B)The number of residual FITC-OS was quantified (n = 8). Scale bar: 20 μm. (C) Compared with NC group, knockdown of *RPGR^ORF15^* made for decreasing number of localizations between the LAMP1 and CTSB in ARPE-19 cells. Scale bar: 20 μm. Scale bar = 10 μm. Data were indicated as mean ± SD. ns, not significant, **p* < 0.05, ***p*<0.01.


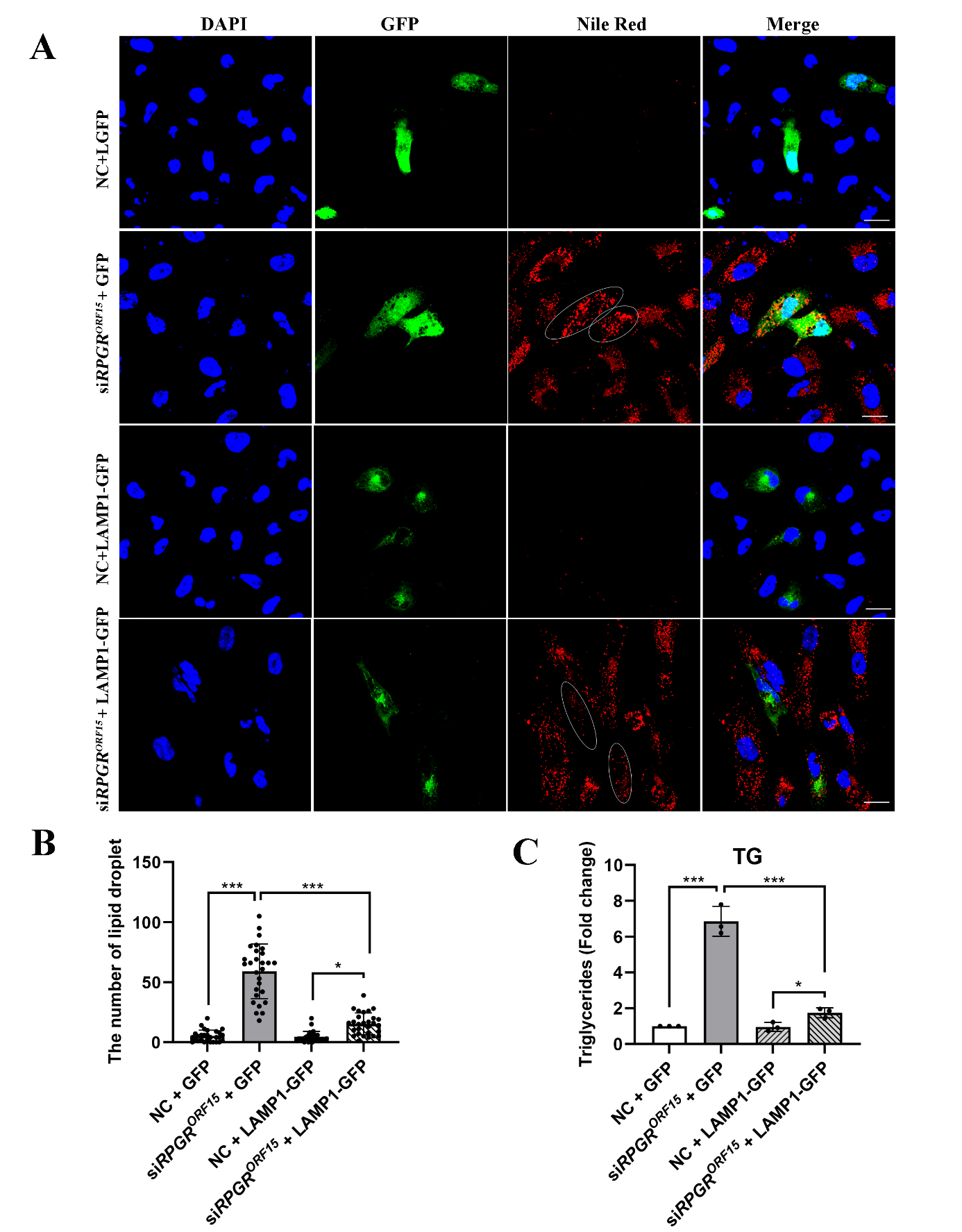


**Figure S8.** Overexpression of *Lamp1* can rescue lipid droplet accumulation in ARPE-19 Cells. (A) Nile red staining in NC + GFP, si*RPGR^ORF15^* + GFP, NC + LAMP1-GFP, si*RPGR^ORF15^* + LAMP1-GFP groups ARPE-19 cells. Scale bar: 20 μm. (B) Quantification of lipid droplets per cell in successfully transfected cells as shown in (A). (n = 28). (C)The triglyceride content was measured between NC + GFP, si*RPGR^ORF15^* + GFP, NC + LAMP1-GFP, si*RPGR^ORF15^* + LAMP1-GFP ARPE-19 cells groups. Three independent experiments were performed with cells from three different passages. Data are presented as mean ± SD. Statistical significance is indicated as ns, not significant, **p* <0.05, ****p* < 0.001.

**Table S1.** List of primary antibodies used in this study.

| Antibody | Source | Recognize | Dilution |
| --- | --- | --- | --- |
| Anti-Rpgra | Customized  from Proteintech | Zebrafish Rpgra | WB: 1:500,  IF: 1:50 |
| Anti-RPGR | Sigma, HPA001593 | human RPGR | WB: 1:1000 |
| Anti-Zo-1 | Genetex, GTX108592 | Zebrafish Zo-1 | IF: 1:100 |
| Anti-Perilipin1 | Abcam, ab172907 | Zebrafish and human Perilipin1 | WB: 1:1000 |
| Anti-Mertk | Santa Cruz, sc-365499 | Zebrafish and human Mertk | WB: 1:1000 |
| Anti-Rab5a | Santa Cruz, sc-46692 | Zebrafish and human Rab5a | WB: 1:1000,  IF: 1:100 |
| Anti-Rab7a | CST, 9367 | Zebrafish and human Rab7a | WB: 1:1000,  IF: 1:100 |
| Anti-CTSB | Proteintech, 12216-1-AP | Human CTSB | WB: 1:1000,  IF: 1:100 |
| Anti-CTSB | Huabio, M1506-1 | Zebrafish CTSB | WB: 1:1000 |
| Anti-LAMP2 | Abcam, ab199946 | Zebrafish and human LAMP2 | WB: 1:800 |
| Anti-LAMP1 | Abcam, ab208943 | Zebrafish LAMP1 | WB: 1:1000 |
| Anti-LAMP1 | CST, 9091 | Human LAMP1 | WB: 1:1000,  IF: 1:100 |
| Anti-LAMP1 | CST, 15665 | Human LAMP1 | IF: 1:100 |
| Anti-Beta Tubulin | Proteintech, 10068-1-AP | Zebrafish and human Tubulin | WB: 1:3000 |
